# Epidemiological association between multiple chemical sensitivity and birth by caesarean section: a nationwide case-control study

**DOI:** 10.1186/s12940-018-0438-2

**Published:** 2018-12-14

**Authors:** Kentaro Watai, Yuma Fukutomi, Hiroaki Hayashi, Yosuke Kamide, Kiyoshi Sekiya, Masami Taniguchi

**Affiliations:** 1 Clinical Research Center for Allergy and Rheumatology, National Hospital Organization Sagamihara National Hospital, 18-1 Sakuradai, Minami-ku, Sagamihara, Kanagawa 252-0392 Japan; 2 Course of Allergy and Clinical Immunology, Juntendo University Graduate School of Medicine, Tokyo, Japan

**Keywords:** Multiple chemical sensitivity, Caesarean section, Case-control study, Epidemiology

## Abstract

**Introduction:**

Multiple chemical sensitivity (MCS) is characterized by recurrent nonspecific symptoms that are attributed to exposure to trace levels of environmental agents. Although the clinical symptoms of MCS have been described in several studies, the risk factors for this condition remain unclear. Our aim was to clarify the risk factors for MCS and the association between MCS and birth by caesarean section.

**Methods:**

We conducted a nationwide case-control study of Japanese individuals (aged 20–65 years) with physician-diagnosed MCS (183 cases) and without MCS (345 controls). The study participants were selected from among 150,000 people in a web-based research panel with approximately 1,000,000 registrants. They completed an online survey including questions on their sociodemographic characteristics, birth history (i.e., birth by caesarean section), and other potential risk factors for MCS. Multivariate logistic regression analysis was employed to determine the association between sociodemographic characteristics and the risk of MCS.

**Results:**

The proportions of case and control subjects who were born by caesarean section were 39.9 and 7.0%, respectively. The association between birth by caesarean section and MCS was significant even after adjusting for potential confounders (adjusted odds ratio: 6.15; 95% confidence interval: 3.13–12.1). A history of agricultural work, mouth breathing, ≥11 vaccinations in the past 10 years, and residing in a new home (< 1 year-old) ≥3 times were also significantly associated with MCS.

**Conclusion:**

Our data indicate an epidemiological link between MCS and birth by caesarean section. Moreover, we show that factors other than chemical exposure may be associated with the development of MCS.

**Electronic supplementary material:**

The online version of this article (10.1186/s12940-018-0438-2) contains supplementary material, which is available to authorized users.

## Background

Individuals with multiple chemical sensitivity (MCS), also called idiopathic environmental intolerance, exhibit nonspecific multi-organ symptoms after exposure to trace amounts of various chemical substances and/or environmental conditions [1, 2]. Although several patient-based studies describe the clinical symptoms of MCS [3, 4], its risk factors remain unclear.

In a telephone survey in the United States, the self-reported prevalence of MCS was 12.6–15.9% [5], whereas the prevalence of physician-diagnosed MCS was 3.1–6.3% [6]. A population-based study in Germany showed self-reported and physician-diagnosed prevalence rates of MCS of 9.6 and 0.5%, respectively [7]. In Japan, a survey by the National Public Health Institute (now the National Health Science Medicine Institute) in 2000 reported that 0.74% of adults had MCS [8].

The aim of this study was to elucidate the risk factors for MCS and the association between MCS and birth by caesarean section. Because MCS is relatively rare in the general population and patients with MCS often avoid hospitals owing to potential exposure to trace amounts of chemicals en route [5–7], we utilized a case-control study design and recruited subjects from a large-scale web-based research panel. We compared sociodemographic characteristics, birth history, medical history, and environmental exposures between patients with and without MCS.

## Methods

### Study design

We performed a case-control study of Japanese residents aged 20–65 years. Cases and controls were selected from a large-scale web-based panel provided by MACROMILL, INC. (Tokyo, Japan). Members of the research panel are voluntary registrants who agreed to answer various web-based surveys for a small fee (membership points). The number of registered members at the time of this study was approximately 1,000,000; this represents 0.8% of the Japanese population. The subjects completed a secure online survey that included questions from the Quick Environmental Exposure and Sensitivity Inventory (QEESI; Additional file 1), as well as questions on their sociodemographic characteristics, environmental exposures, and medical histories.

The ethics committee of the Sagamihara National Hospital approved the study protocol (No. 150912, approved on September 15, 2015) in accordance with the Declaration of Helsinki. The study participants’ informed consents were obtained when they registered for the web-based study.

### Assessment of multiple chemical sensitivity

The QEESI is the most widely used tool for evaluating MCS. It is both sensitive (92%) and specific (95%) for MCS [9–13], and the reliability and validity of the Japanese version of the QEESI have been confirmed [9].

We used the QEESI and a self-reported history of physician-diagnosed MCS to select patients with MCS. The QEESI consists of 50 questions divided into five sections: I) chemical exposures, II) other exposures, III) symptoms, IV) masking index, and V) impact of sensitivities. All sections except IV) (masking index) are scored with a total of 0 to 100 points. MCS risk is based on chemical exposures (section I) and symptoms (section III). Individuals with a total score ≥ 40 in each of these sections are defined as ‘very suggestive of MCS’ [11], whereas those with a total score < 40 are defined as ‘not suggestive of MCS’. We included questions in sections I) and III) in our web-based survey.

### Web-based survey

#### Primary survey

**Fig. 1:**
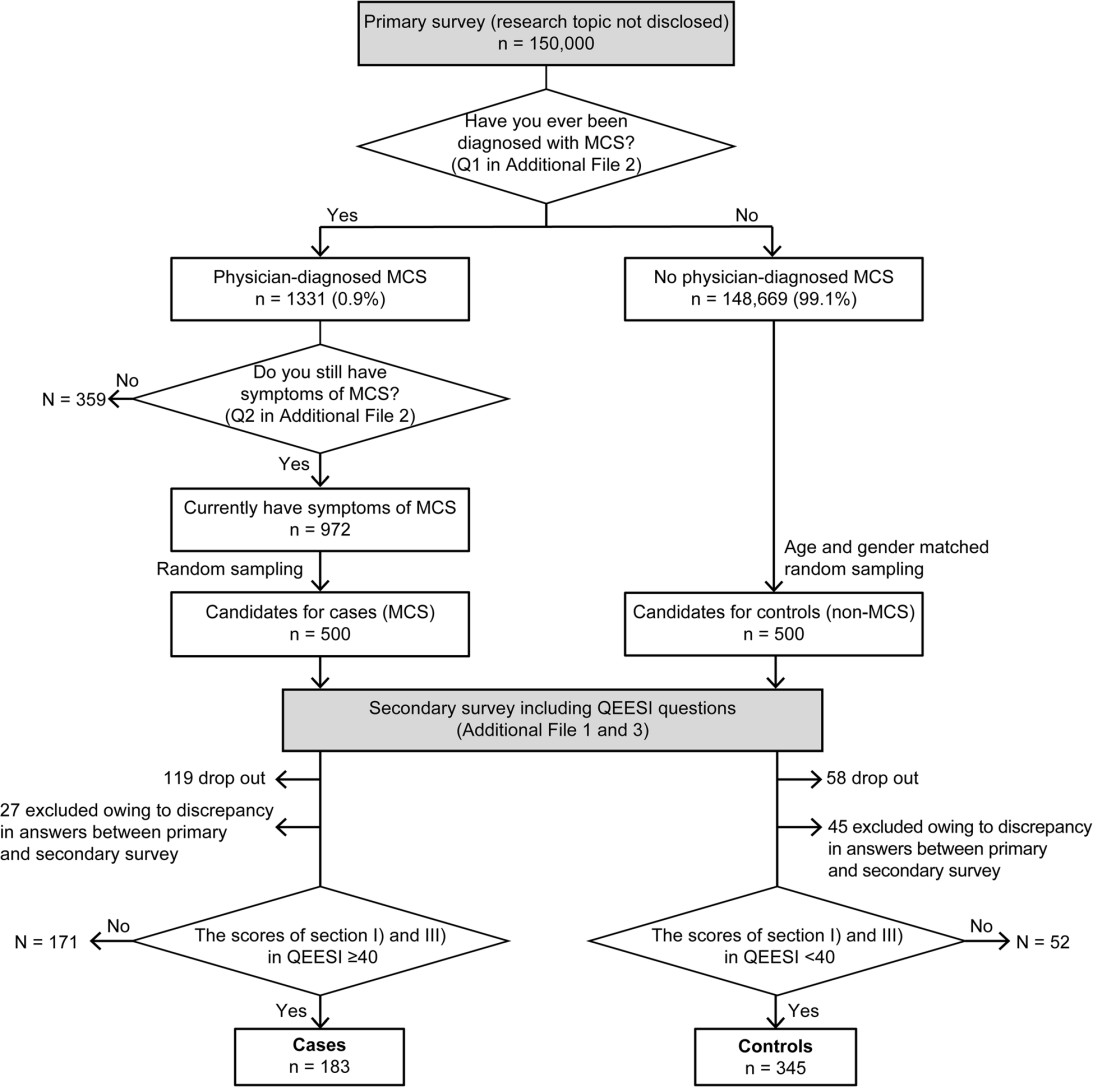
Protocol used for the web-based survey. MCS, multiple chemical sensitivity; QEESI, Quick Environmental Exposure and Sensitivity Inventory

Figure 1 shows the protocol used for the web-based survey. Between March 17 and March 27, 2016, MACROMILL INC. sent emails inviting randomly selected, age-stratified research panel registrants to participate in a screening survey. Once 150,000 responses had been obtained, no further invitations were sent. The initial web-based screening questionnaire consisted of three questions (Qs) on MCS (Q1 to Q3) (Additional file 2). Subjects were considered symptomatic physician-diagnosed MCS cases if they met both of the following two criteria: (i) indicated ‘MCS’ in the response to Q1, ‘Which of the diseases listed below have you ever been diagnosed with?’ and (ii) provided an affirmative response to Q2, ‘Do you have symptoms of MCS now?’ Q2 was only asked to subjects who indicated ‘MCS’ in Q1. Q3 asked if patients were born by caesarian section. For the secondary survey, 500 candidates were randomly selected from among the 972 subjects who fulfilled the two criteria. As controls, 500 age- and sex-matched candidates were chosen from among those who did not indicate ‘MCS’ in the response to Q1. Consequently, email invitations to the secondary survey were sent to 500 cases and 500 controls.

#### Secondary survey

Email invitations to the secondary survey were sent from March 29 to March 31, 2016; reminder emails were sent up to three times. The secondary survey contained detailed questions from sections I) and III) of the Japanese version of the QEESI (Additional file 1) as well as questions on the subjects’ sociodemographic characteristics (Additional file 3). It also included questions on the potential risk factors for MCS, namely, comorbidities of allergic diseases, chemical exposures, occupational history, history of vaccinations, number of times of living in a house within 1 year of its construction, birth history, and family history of MCS (Additional file 3).

To ensure the validity of the answers obtained in the primary survey, the secondary survey also included Q1 and Q2 of the primary survey. Of the 500 cases who received an invitation to the secondary survey, 381 responded (response rate, 76%). We excluded 27 cases with discrepancies in the answers to Q1 and Q2 between the primary and secondary surveys, as well as 171 cases with total scores of < 40 each in sections I) and III). Of the 500 controls who received an invitation to the secondary survey, 442 responded (response rate, 88%). We excluded 45 controls with discrepancies in the answers to Q1 and Q2 between the primary and secondary surveys, as well as 52 controls with total scores of ≥40 each in sections I) and III). Finally, data from 183 cases and 345 controls were included in the analyses.

### Cases and controls

To summarize, the cases (MCS patients) in our study (i) were diagnosed with MCS by a physician, (ii) currently had MCS symptoms, and (iii) were classified as ‘very suggestive of MCS’ in the QEESI (chemical exposures and symptoms scores both ≥40) [11]. The controls in our study (i) had never been diagnosed with MCS by a physician, (ii) were age-and sex-matched to the cases, and (iii) were classified as ‘not suggestive of MCS’ (chemical exposures and symptoms scores both < 40) [11]. The number of cases and controls was 183 and 345, respectively.

### Statistical analysis

All data were analyzed using SPSS version 22.0 software (IBM, Tokyo, Japan). Categorical variables were compared using the chi-squared or Fisher’s exact test, and continuous variables were compared using the T-test or Mann-Whitney U test. Multivariate logistic regression was employed to compare the sociodemographic characteristics of cases and controls and to determine the adjusted odds ratios and 95% confidence intervals for the association between sociodemographic characteristics and the risk of MCS after adjustments for sex, age, history of smoking, bronchial asthma, and allergic rhinitis. In order, the reasons for these adjustments were as follows: more women have MCS than men; the number of times a person resides in a newly built house is influenced by age; tobacco smoke, a pervasive environmental pollutant, worsens MCS symptoms; and bronchial asthma and allergic rhinitis are common in MCS patients [5]. A *P* value < 0.05 was considered statistically significant.

## Results

The prevalence of physician-diagnosed MCS in the 150,000 individuals in the primary survey was 0.9% (Table 1). It was significantly higher in men than women in the younger age groups (20–35 years) and significantly higher in women than men in the older age groups (41–65 years).

**Table 1:** Physician-diagnosed multiple chemical sensitivity according to sex and age in the primary survey

Age	Men (*N* = 64,675)	Women (*N* = 85,325)	Total (*N* = 150,000)
20–25 years*	35/1713	64/6362	99/8075
	2.0 (1.37–2.71)	1.0 (0.76–1.25)	1.2 (0.99–1.47)
26–30 years*	56/2746	71/11,474	127/14,220
	2.0 (1.51–2.57)	0.6 (0.48–0.76)	0.9 (0.74–1.05)
31–35 years*	74/4529	115/13,769	189/18,298
	1.6 (1.26–2.00)	0.8 (0.68–0.99)	1.0 (0.89–1.18)
36–40 years	59/6816	117/13,079	176/19,895
	0.9 (0.65–1.09)	0.9 (0.73–1.06)	0.9 (0.75–1.01)
41–45 years*	60/10,311	166/13,181	226/23,492
	0.6 (0.44–0.73)	1.3 (1.07–1.45)	1.0 (0.84–1.09)
46–50 years*	64/10,880	95/10,222	159/21,102
	0.6 (0.44–0.73)	0.9 (0.74–1.12)	0.8 (0.64–0.87)
51–55 years*	62/10,921	93/7748	155/18,669
	0.6 (0.43–0.71)	1.2 (0.96–1.44)	0.8 (0.70–0.96)
56–60 years*	45/8672	61/5277	106/13,949
	0.5 (0.37–0.67)	1.2 (0.87–1.44)	0.8 (0.62–0.90)
61–65 years^*^	49/8087	45/4213	94/12,300
	0.6 (0.44–0.78)	1.1 (0.76–1.38)	0.8 (0.61–0.92)
Total	504/64,675	827/85,325	1331/150,000
	0.8 (0.71–0.85)	1.0 (0.90–1.03)	0.9 (0.84–0.93)

The values in the upper rows represent the number of subjects with multiple chemical sensitivity/the total number of subjects. The values in the lower rows are the percentages (95% confidence intervals)

*P < 0.01, significant difference between men and women using the chi-squared test

The sociodemographic characteristics of the 183 cases (subjects with MCS) and 345 controls are shown in Table 2. The questionnaire used to collect these data is described in Additional file 3. The proportion of subjects with a history of smoking was significantly higher in the case group than the control group, as was the incidence of bronchial asthma, allergic rhinitis, metal allergy, fibromyalgia, chronic fatigue syndrome, electromagnetic hypersensitivity, and migraine. The mean age of onset of MCS was 23.6 ± 12.1 years.

**Table 2:** Sociodemographic characteristics of cases and controls

Variable	Cases (MCS) *n* = 183	Controls (non-MCS) *n* = 345	*P* value
Age (years), mean ± SD	39.8 ± 10.6	40.2 ± 10.9	0.853^‡^
Age at MCS onset (years), mean ± SD	23.6 ± 12.1	NA	
Female sex, n (%)	109 (59.6)	200 (58.0)	0.724
Body mass index, median (IQR)	21.1 (19.0–23.0)	21.3 (19.5–23.9)	0.104^§^
History of smoking, n (%)	103 (56.3)	127 (36.8)	< 0.001
Age at start of smoking (years), mean ± SD	21.0 ± 6.12	19.2 ± 3.20	0.008^‡^
Passive smoking, n (%)	106 (57.9)	125 (36.2)	< 0.001
Birth by caesarean section, n (%)	73 (39.9)	24 (7.0)	< 0.001
History of MCS			
Patient’s mother, n (%)	31 (16.9)	1 (0.3)	< 0.001
Patient’s father, n (%)	20 (10.9)	0(0)	< 0.001
Patient’s sibling(s), n (%)	35 (19.1)	3 (0.9)	< 0.001
Comorbidity, n (%)			
Bronchial asthma	48 (26.2)	9 (2.6)	< 0.001
Allergic rhinitis	119 (65.0)	78 (22.6)	< 0.001
Metal allergy	76 (41.5)	9 (2.6)	< 0.001
Fibromyalgia	22 (12.0)	0 (0)	< 0.001
Chronic fatigue syndrome	25 (13.7)	0 (0)	< 0.001
Electromagnetic hypersensitivity	25 (13.7)	0 (0)	< 0.001
Migraine	62 (33.9)	46 (13.3)	< 0.001
Occupational history, n (%)			
Worker in the manufacturing industry	43 (23.5)	57 (16.5)	0.052
Construction worker	14 (7.7)	13 (3.8)	0.054
Agricultural worker	19 (10.4)	5 (1.4)	< 0.001
Chemical researcher	6 (3.3)	4 (1.2)	0.089^¶^
Cosmetics salesperson	10 (5.5)	3 (0.9)	0.001^¶^
Shoe store clerk	10 (5.5)	2 (0.6)	< 0.001^¶^
Healthcare worker	22 (12)	23 (6.7)	0.036
Drugstore clerk	10 (5.5)	5 (1.4)	0.008
Pet ownership before MCS onset^†^	146 (79.8)	203 (58.8)	< 0.001
Mouth breathing	126 (68.9)	140 (40.6)	< 0.001
Number of vaccinations in the past 10 years, n (%)			< 0.001
0	48 (26.2)	162 (47.0)	
1–5	49 (26.8)	111 (32.2)	
6–10	32 (17.5)	56 (16.2)	
≥ 11	54 (29.5)	16 (4.6)	
Number of times living in a house < 1 year-old, n (%)			< 0.001
0	40 (21.9)	138 (40.0)	
1–2	114 (62.3)	198 (57.4)	
≥ 3	29 (15.8)	9 (2.6)	

†Pets include dogs, cats, hamsters, rabbits, guinea pigs, ferrets, and birds but not animals kept for commercial purpose. ^‡^T-test. ^§^Mann-Whitney U test. ^¶^Fisher’s exact test. No symbol, chi-squared test. IQR, interquartile range; MCS, multiple chemical sensitivity; NA, not applicable; SD, standard deviation

Birth by caesarean section correlated with MCS. The proportions of subjects who were born by caesarean section were 39.9 and 7.0% in the case and control groups, respectively (*P* < 0.001). We found a significant association between a family history of MCS and MCS (Table 2). Birth by caesarean section remained significantly associated with MCS in a multivariate logistic regression analysis adjusted for sex, age, history of smoking, bronchial asthma, and allergic rhinitis (adjusted odds ratio: 6.15; 95% confidence interval: 3.13–12.1) (Table 3).

**Table 3:** Risk factors for multiple chemical sensitivity in a multivariate logistic regression analysis

Variable	Adjusted† OR (95% CI)	P value
Birth by caesarean section	6.15 (3.13–12.1)	< 0.001
Occupational history		
Agricultural worker	4.79 (1.31–17.6)	0.018
Cosmetics salesperson	2.31 (0.43–12.5)	0.332
Shoe store clerk	2.46 (0.38–16.0)	0.348
Healthcare worker	1.95 (0.84–4.50)	0.119
Drugstore clerk	2.56 (0.54–12.1)	0.236
Pet ownership before MCS onset^‡^	1.56 (0.90–2.69)	0.115
Mouth breathing	1.69 (1.02–2.80)	0.043
Number of times of living in a house < 1 year-old	4.63 (1.54–14.0)	0.006
0	1	
1–2	0.96 (0.55–1.67)	0.880
≥ 3	4.29 (1.34–13.7)	0.014
Number of vaccinations in the past 10 years		
0	1	
1–5	1.26 (0.69–2.31)	0.446
6–10	1.37 (0.67–2.80)	0.383
≥ 11	5.33 (2.19–12.9)	< 0.001

^†^Adjusted for sex, age, history of smoking, bronchial asthma, and allergic rhinitis

^‡^Pets include dogs, cats, hamsters, rabbits, guinea pigs, ferrets, and birds but not animals kept for commercial purposes

*CI* confidence interval, *MCS* multiple chemical sensitivity, *OR* odds ratio

Social/environmental factors also significantly correlated with MCS. As determined via multivariate regression analysis adjusted for the variables listed above, these factors were a history of agricultural work, mouth breathing, residing in a new home (within 1 year of its construction) ≥3 times, and ≥ 11 vaccinations in the past 10 years (Table 3).

## Discussion

To the best of our knowledge, our study is the first to show an epidemiological link between MCS and birth by caesarean section in Japan. Moreover, our data indicate that factors other than chemical exposure may result in the development of MCS.

The prevalence of MCS among the individuals in our study was 0.9%. Interestingly, MCS was significantly more prevalent in men than women in the younger age groups but significantly higher in women than men in the older age groups. The reason for this age-based sex difference remains unknown. Although the prevalence of MCS is generally higher in women than men [5, 6], no inversion of the sex predominance between younger and older age groups has been reported. The prevalence of MCS in this study is lower than that reported in a large-scale survey in the United Sates [5, 6], but about the same as that reported in surveys in Germany [7] and Japan [8].

In our study, the proportion of subjects with a history of smoking was significantly higher in the case (MCS) group than the control group. Although patients with MCS are sensitive to environmental tobacco smoke (ETS) [5], their smoking history and ETS exposure did not correlate with the development of MCS in a previous study [14]. Hence, the association between smoking and MCS requires further research.

Our finding that comorbidities such as allergic diseases were significantly associated with MCS is in agreement with the findings of a survey in the United States [5]. Central sensitization, which involves hyper-excitation of the neurons in the central nervous system due to stimulation from the peripheral nervous system [15, 16], has been suggested as a biopsychological explanation for MCS [17, 18]. MCS, fibromyalgia, chronic fatigue syndrome, and migraine are all considered central sensitization syndromes [17]. Therefore, the association between MCS and comorbidities in this study is likely related to central sensitization.

Birth by caesarean section significantly correlated with MCS in our study even after adjustments for potential confounders. This finding adds to the list of conditions associated with caesarean section. For example, two meta-analyses in 2008 showed a 20% higher asthma risk in children delivered by caesarean section than in those delivered vaginally [19, 20]. Although the mechanism is unknown, mothers with MCS are at increased risk of having a child with autism spectrum disorders (ASD) or attention deficit hyperactivity disorder (ADHD) [21]. Infants born by caesarean section are not exposed to the bacteria in the birth canal, which can cause abnormalities in the gut microbiome [22–24]. Owing to the brain-gut interaction, such abnormalities can lead to brain dysfunction [25–27] and perhaps contribute to the development of MCS. Brain sensitivity and central sensitization may also be associated with MCS [17, 18, 28]; central sensitization enhances the activity of the neurons involved in nociception, resulting in hypersensitivity to stimuli [29, 30]. Abnormalities in the gut microbiome caused by birth by cesarean section might therefore contribute to the development of MCS. Moreover, unlike vaginal delivery, a caesarean section may indirectly expose a fetus or newborn to surgical chemicals (e.g., analgesics and anesthetics administered to the mother). Understanding the mother’s background and reasons for receiving a caesarean section is necessary to evaluate the influence of caesarean sections on MCS.

Mouth breathing was associated with MCS in our adjusted regression model. Adaption to olfactory hypersensitivity, a characteristic of MCS, may account for mouth breathing in MCS patients [31]. However, the likely association of bronchial asthma and allergic rhinitis with MCS indicates that upper and lower respiratory tract diseases may trigger MCS [5]. Mouth breathing bypasses the protective functions of the nose such as warming, humidifying, and filtering the air [32, 33]. Studies with small sample sizes reported that mouth breathing adversely affected lung function and caused exercise-induced bronchoconstriction in patients with mild asthma [34] and that enforced mouth breathing impaired lung function [35]. Although the causal relationship between mouth breathing and MCS is not clear, it is possible that mouth breathing initiates or exacerbates MCS via comorbidities such as bronchial asthma and allergic rhinitis.

Having been vaccinated ≥11 times in the past 10 years was significantly associated with MCS in our adjusted regression model. Although it would have been ideal to investigate the subjects’ entire vaccination history, we considered the recall bias to be large and therefore limited the vaccination history to the last 10 years. To reduce the occurrence of infectious diseases, regular vaccination during childhood is recommended. However, the Vaccine Safety Datalink study showed a significant association between exposure to high levels of mercury in thimerosal-containing children’s vaccines and the subsequent risk of atypical autism [36]. Thimerosal is an organomercury preservative that is often added, even today, to multiple-dose vials of many vaccines. In a previous case-control study, maternal chemical intolerance correlated with attention deficit hyperactivity disorder (ADHD) and autism spectrum disorder (ASD) in the offspring (282 AHD cases, 258 ADHD cases, and 154 cases without these disorders) [21]. Although it is not clear how, vaccination and MCS might be linked.

Having lived ≥3 times in a new house (i.e., a house < 1 year-old) was significantly associated with MCS in our adjusted regression model. Sick building syndrome (SBS) is defined as occupancy-dependent nose, eye, and skin irritations: the symptoms occur within a building but disappear or weaken outside the building. SBS is grouped under the more general definition of MCS/idiopathic environmental intolerance [37]. Indoor environmental factors in a newly built house like inadequate ventilation, high total levels of volatile organic compounds, and dampness could be risk factors for both SBS [38–41] and MCS.

Having been an agricultural worker was significantly associated with MCS in our adjusted regression model. Such workers are exposed to pesticides [42] when working in recently sprayed fields as well as during the preparation and application of the pesticide [43]. Pesticide exposure is a major health hazard that can lead to various illnesses including respiratory disorders [44–49]. Although the mechanism remains unknown, MCS is often complicated by upper and lower respiratory tract diseases [5]. Exposure to pesticides during agricultural work can affect a worker’s respiratory tract and might cause the development of MCS.

This study has several limitations. First, MCS was defined through self-reported physician-diagnosis. However, we ascertained the history of the physician diagnosis by obtaining the name of the hospital or clinic where the MCS diagnosis was made. Moreover, self-reported and actual physician diagnoses have been shown to correlate significantly [5]. Second, no objective measurement was used for diagnosing MCS. Although some studies have identified abnormalities in cerebral blood flow as well as mutations in several genes and metabolic enzymes in MCS [50–53], these are not considered evidence-based indicators of MCS at present. We used the QEESI to define patients with MCS as this tool has been internationally validated [9, 11–13]. Third, we may have overlooked individuals who do not typically access the web; we note that most MCS patients have electromagnetic hypersensitivity [54], which prevents them from using a personal computer. However, some MCS patients have difficulties visiting healthcare facilities owing to potential exposure to chemical substances (such as perfumes and exhaust gases) while on route. A web-based survey can overcome this limitation as the patients do not need to leave their homes.

The main strength of this study was its use of a sizeable web-based survey to identify cases and controls in the general population. Patients with rare diseases are identifiable only in large-scale surveys.

## Conclusions

In conclusion, this study showed an epidemiological link between MCS and birth by caesarean section. Our data also suggest that factors other than chemical exposure may result in the development of MCS.

## Additional files

Additional file 1: Quick Environmental Exposure and Sensitivity Inventory (QEESI) (DOCX 16 kb)

Additional file 2: Screening questionnaire (DOCX 15 kb)

Additional file 3: Questionnaire for the secondary survey (DOCX 22 kb)

## Abbreviations

ADHD: Attention deficit hyperactivity disorder
ASD: Autism spectrum disorder
ETS: Environmental tobacco smoke
MCS: Multiple chemical sensitivity
Q: Question
QEESI: Quick Environmental Exposure and Sensitivity Inventory
SBS: Sick building syndrome
