# Supplementary material for: Epidemiological association between multiple chemical sensitivity and birth by caesarean section: a nationwide case-control study
Source: Environ Health. 2018 Dec 14;17:89. doi: 10.1186/s12940-018-0438-2 (PMC6295056; doi:10.1186/s12940-018-0438-2)
Supplement: Supplementary file 1 — Quick Environmental Exposure and Sensitivity Inventory (QEESI) (DOCX 16 kb) [file 12940_2018_438_MOESM1_ESM.docx]

***Additional File 1***

***Quick Environmental Exposure and Sensitivity Inventory (QEESI)***

The QEESI is a validated tool for evaluating chemical intolerance [1, 2]. It is self-administered and consists of five sections: I) chemical exposures, II) other exposures, III) symptoms, IV) masking index, and V) impact of sensitivities. Sections I) and II) assess the severity of the exposure, and sections III) and IV) assess the severity of the symptom. Each section contains 10 items scored as follows: 0 = not a problem, 5 = moderate, and 10 = severe or disabling. The scores of the 10 items are combined, and a total score of 0 to 100 is obtained for each section. The Japanese version of the QEESI has been validated [3].

*Chemical exposures scale (section I)*

This section assesses the severity of the response to 10 commonly inhaled agents: diesel or gas engine exhaust, tobacco smoke, insecticides/herbicides, gasoline vapors, paint/paint thinners, cleaning products/disinfectants, fragrances/aromatics, fresh tar/asphalt, nail polish/nail polish remover/hairspray, and new furnishings/new cars.

*Symptoms scale (section III)*

This section assesses symptoms in multiple organs or regions: musculoskeletal; mucus membranes of the eyes, nose, and respiratory system; heart/chest; gastrointestinal; head; neuromuscular; skin; and genitourinary. Cognitive function and emotions are also examined.

*References*

1. Miller CS, Prihoda TJ. A controlled comparison of symptoms and chemical intolerances reported by Gulf War veterans, implant recipients and persons with multiple chemical sensitivity. Toxicol Industr Health. 1996:15;386–97.

2. Miller CS, Prihoda TJ. The Environmental Exposure and Sensitivity Inventory (EESI): a standardized approach for measuring chemical intolerances for research and clinical applications. Toxicol Industr Health. 1996:15;370–85.

3. Hojo S, Kumano H, Yoshino H, Kakuta K, Ishikawa S. Application of Quick Environment Exposure Sensitivity Inventory (QEESI) for Japanese population: study of reliability and validity of the questionnaire. Toxicol Industr Health. 2003:19;41–9.
