# Supplementary material for: Epidemiological association between multiple chemical sensitivity and birth by caesarean section: a nationwide case-control study
Source: Environ Health. 2018 Dec 14;17:89. doi: 10.1186/s12940-018-0438-2 (PMC6295056; doi:10.1186/s12940-018-0438-2)
Supplement: Supplementary file 2 — Screening questionnaire (DOCX 15 kb) [file 12940_2018_438_MOESM2_ESM.docx]

***Additional File 2***

***Screening questionnaire***

Q1 Which of the diseases listed below have you ever been diagnosed with? Mark all choices that apply.

Asthma; Allergic rhinitis; Atopic dermatitis; Multiple chemical sensitivity; Irritable bowel syndrome; Migraine; Diabetes mellitus; Hypertension; Hyperlipidemia; Sinusitis; Allergic conjunctivitis

(Condition: Answer question Q2 only if you chose ‘Multiple chemical sensitivity’ in Q1.)

Q2 Do you still have symptoms of multiple chemical sensitivity?

Yes/No

(Condition: All subjects who answered Q1.)

Q3 Were you born by caesarean section?

Yes/No
