# Supplementary material for: Epidemiological association between multiple chemical sensitivity and birth by caesarean section: a nationwide case-control study
Source: Environ Health. 2018 Dec 14;17:89. doi: 10.1186/s12940-018-0438-2 (PMC6295056; doi:10.1186/s12940-018-0438-2)
Supplement: Supplementary file 3 — Questionnaire for the secondary survey (DOCX 22 kb) [file 12940_2018_438_MOESM3_ESM.docx]

***Additional File 3***

***Questionnaire for the secondary survey (the descriptions in parentheses correspond to the variables in Table 2)***

Q1 (Age at MCS onset)

How old were you when you first experienced chemical hypersensitivity?

Q2 (Body mass index)

How tall are you? How much do you weigh?

Body mass index is calculated as weight in kilograms divided by height in meters squared.

Q3 (History of smoking)

Have you ever smoked for at least 1 year?

Yes: at least one cigarette per day or one cigar a week on average during a 1-year period.

No: less than one cigarette per day or one cigar per week on average during a 1-year period.

(Condition: answer question Q4 only if you chose ‘Yes’ in Q3.)

Q4 (Age at start of smoking)

How old were you when you started smoking? Even if you began smoking when you were less than 20 years-old, please enter the specific age.

Q5 (Passive smoking)

This is a question about passive smoking. Is there a person who routinely smokes in the same room in your home or workplace?

Yes/no

Q6 (History of MCS)

Are members of your family hypersensitive to multiple chemicals? If you have more than one sibling, please choose the one closest to you in age when answering the question.

Yes/No for father, mother, and sibling

Q7 (Comorbidity)

Which of the diseases listed below have you ever been diagnosed with? Mark all choices that apply.

Bronchial Asthma; Allergic rhinitis; Metal allergy; Fibromyalgia; Chronic fatigue syndrome; Electromagnetic hypersensitivity; Migraine

Q8 (Occupational history)

Which of the following jobs have you held? Mark all choices that apply.

Worker in the manufacturing industry; Construction worker; Agricultural worker; Chemical researcher; Cosmetics salesperson; Shoe store clerk; Healthcare worker; Drugstore clerk

Q9 (Pet ownership before onset)

Did you have any pets before developing chemical hypersensitivity? Pets includes dogs, cats, hamsters, rabbits, guinea pigs, ferrets, and birds but not animals kept for commercial purposes.

Yes/No

Q10 (Mouth-breathing)

Do you often breathe through your mouth while awake or sleeping?

Yes/No

Q11 (Number of vaccinations in the past 10 years)

How many vaccinations have you received in the last 10 years?

0/1–5/6–10/11 or more

Q12 (Number of times living in a house less than 1 year-old)

How many times have you lived in a house within 1 year of its construction?

0/1–2/3 or more
